# The Development and Evaluation of a Loop-Mediated Isothermal Amplification Method for the Rapid Detection of *Salmonella enterica serovar* Typhi

**DOI:** 10.1371/journal.pone.0124507

**Published:** 2015-04-24

**Authors:** Fenxia Fan, Pengcheng Du, Biao Kan, Meiying Yan

**Affiliations:** 1 State Key Laboratory for Infectious Disease Prevention and Control, National Institute for Communicable Disease Control and Prevention, Chinese Center for Disease Control and Prevention, Beijing, China; 2 Collaborative Innovation Center for Diagnosis and Treatment of Infectious Diseases, Hangzhou, Zhejiang, China; Panjab University, INDIA

## Abstract

Typhoid fever remains a public health threat in many countries. A positive result in traditional culture is a gold-standard for typhoid diagnosis, but this method is time consuming and not sensitive enough for detection of samples containing a low copy number of the target organism. The availability of the loop-mediated isothermal amplification (LAMP) assay, which offers high speed and simplicity in detection of specific targets, has vastly improved the diagnosis of numerous infectious diseases. However, little research efforts have been made on utilizing this approach for diagnosis of *Salmonella enterica serovar* Typhi by targeting a single and specific gene. In this study, a LAMP assay for rapid detection of *S*. Typhi based on a novel marker gene, termed STY2879-LAMP, was established and evaluated with real-time PCR (RT-PCR). The specificity tests showed that STY2879 could be amplified in all *S*. Typhi strains isolated in different years and regions in China, whereas no amplification was observable in non-typhoidal strains covering 34 *Salmonella* serotypes and other pathogens causing febrile illness. The detection limit of STY2879-LAMP for *S*. Typhi was 15 copies/reaction in reference plasmids, 200 CFU/g with simple heat-treatment of DNA extracted from simulated stool samples and 20 CFU/ml with DNA extracted from simulated blood samples, which was 10 fold more sensitive than the parallel RT-PCR control experiment. Furthermore, the sensitivity of STY2879-LAMP and RT-PCR combining the traditional culture enrichment method for simulated stool and blood spiked with lower *S*. Typhi count during the 10 h enrichment time was also determined. In comparison with LAMP, the positive reaction time for RT-PCR required additional 2-3 h enrichment time for either simulated stool or blood specimens. Therefore, STY2879-LAMP is of practical value in the clinical settings and has a good potential for application in developing regions due to its easy-to-use protocol.

## Introduction

Typhoid fever, which is caused by the *Salmonella enterica serovar* Typhi, remains a public health threat in many countries particularly those with poor sanitary conditions. It was estimated that 21 million new cases appear and 216,000 deaths are caused by typhoid fever each year worldwide [1]. Patients with typhoid fever have a non-specific presentation that is similar with other febrile illnesses such as *S*. Paratyphi A, *Leptospirosis*, and *Streptococcus pneumoniae* infection [2, 3], highlighting the importance of the etiological diagnosis of this disease. Bacterial culture is commonly used for diagnosis of typhoid fever and is considered a gold standard of diagnosis, but is time-consuming (at least 3–5 days) and has low sensitivity (30–60%) [4, 5]. A rapid, simple and inexpensive technique for specific diagnosis of *S*. Typhi infection is still needed especially in the regions with high incidence of typhoid.

Several immunological methods including Widal test, Typhi-Dot, Tubex [6], lateral flow [7] and SPR [8] based on the types of O and H antigens have been developed for detection of *S*. Typhi. Despite their simplicity and rapidity, the sensitivity of these assays are relatively low and prone to be influenced by the process of the disease, the judgment of the immunological reaction depends on the antibody production and its titer, which is usually induced at least one week after *S*. Typhi infection, preventing early diagnosis by this approach. In addition, conflicting results in terms of specificity and sensitivity of these serological tests were presented in different typhoid endemic areas [6, 9–13]. The technique of SPR for detection of *S*. Typhi needs further validation in filed. The nucleic acid amplification tests with the advantages of being rapid, specific and sensitive, have been applied to diagnosing *S*. Typhi by targeting certain genes [14–19]. However, these techniques have not been widely applied due to the major limitation of the requirement of a thermal cycler and skilled technicians.

Recently a novel nucleic acid amplification method termed the Loop-mediated isothermal amplification (LAMP) was reported and widely used for the detection of pathogens in clinical diagnostics as a rapid, accurate and cost-effective method [20, 21]. This method can amplify DNA efficiently (up to 10^9^ target DNA copies), specifically and quickly (within 60 min) under isothermal conditions without an obvious interference of the co-existing non-target DNA. The LAMP reaction is easy to perform and requires only suitable primers, Bst polymerase (a strand-displacing DNA polymerase) and a water bath, which make the assay a potentially rapid and simple detection tool for diagnosis of *S*. Typhi infection; this afterwards can promote the management and control of typhoid and reduce the incidence.

The specificity of LAMP assay was dependent on the choice of the target genes. In this study, *S*. Typhi gene STY2879 which encodes a reverse transcriptase family protein was selected as the target, as it is conserved in all tested *S*. Typhi isolates, and not detected in non-typhoidal strains. Therefore, the purpose of this study was to develop a LAMP assay with high specificity and sensitivity to detect *S*. Typhi infection based on STY2879 gene. We then evaluated the performance with pathogen-simulated human blood and stool samples.

## Materials and Methods

### Ethics statement

Blood and feces samples were acquired with the written informed consent from healthy donors who worked in the National Institute for Communicable Disease Control and Prevention, China CDC during annual healthy physical examinations. This study was reviewed and approved by the ethics committee of the National Institute for Communicable Disease Control and Prevention, China CDC, according to the medical research regulations of the Ministry of Health, China (Approval No. ICDC2014003).

### Strains

A total of 97 *Salmonella* strains were used for the specificity testing, including 22 *S*. Typhi and 75 non-typhoidal *Salmonella* isolates, as described in Table 1. These strains cover 35 serotypes. All of the serotypes of *Salmonella* were identified by the Danish Salmonella antisera. In addition, the strain panel also contains other enteric pathogens and those febrile pathogens isolated from blood, including *Staphylococcus aureus*, *Streptococcus pneumoniae*, *Borrelia burgdorferi*, *Leptospira*, *Legionella pneumophila*, *Neisseria meningitidis*, *Rickettsia*, and *Brucella monocytogenes*. All of these strains were obtained came from the National Institute for Communicable Disease Control and Prevention, Chinese Center for Disease Control and Prevention.

**Table 1:** Pathogenic strains used in the STY2879-LAMP assay.

*Salmonella* spp.	LAMP RT-PCR	*Salmonella* spp.	LAMP RT-PCR	Other Pathogens	LAMP RT-PCR
*S*. Typhi(22)*	+	*S*. Pomona (1)	-	**Enteric pathogens**	-
*S*. Paratyphi A(4)	**-**	*S*. Potsdam(1)	-	*V*. *cholerae* serogroup O1(2)	-
*S*. Paratyphi B(2)	-	*S*. Mbandaka(1)	-	*V*. *cholerae* serogroup O139(2)	-
*S*. Paratyphi C(1)	-	*S*. Istanbul(1)	-	*V*. *parahaemolyticus*(4)	-
*S*. Cholera suis(2)	-	*S*. Hvittingfoss(1)	-	*Shigella dysenteriae*(1)	-
*S*. Typhimurium(2)	-	*S*. Litchfield(1)	-	*Shigella flexneri* (1)	-
*S*. Enteritidis(2)	-	*S*. Indiana(1)	-	*Shigella boydii* (1)	-
*S*. Derby(2)	-	*S*. Gateshead(1)	-	*Shigella sonnei* (ATCC25931)	-
*S*. Thompson(2)	-	*S*. Virchow(1)	-	Enterotoxigenic *Escherichia coli* (1)	-
*S*. Senftenberg(2)	-	*S*. Wilhelmsburg(1)	-	Enterohemorrhage *Escherichia coli* (EDL933)	-
*S*. Weltevreden(2)	-	*S*. Wandsworth(1)	-		
*S*. Agona(2)	-	*S*. Schwarzengrund(1)	-	**Other febrile pathogens**	-
*S*. Aberdeen(2)	-	*S*. Livingston(1)	-	*Staphylococcus aureus*(5)	-
*S*. Anatum(1)	-	*S*. Liverpool(1)	-	*Klebsiella pneumonia* (ATCC700603)	-
*S*. Meleagridis(1)	-	*S*. Stanlyville(1)	-	*Borrelia burgdorferi*(ATCC35210)	-
*S*. Sandiego(1)	-			*Leptospira*(ATCC43642)	-
*S*. Uganda(1)	-			*Legionella*(ATCC33153)	-
*S*. Kentukey(1)	-			*Neisseria meningitidis*(1)	-
*S*. Montevideo(1)	-			*Rickettsia* (ATCCVR-142)	-
*S*. Stanly(1)	-			*Brucella*(ATCC23456 ATCC23448 ATCC23444)	-
				*Streptococcus pneumoniae* (ATCC496191)	-

*(n) “n” is the number of the strain used in the study.

### Pathogen-simulated blood specimen preparation and DNA extraction

Fresh human blood was obtained from healthy donors during healthy physical examinations and tested by the culture method and RT-PCR [22] to detect *Salmonella*. No *S*. Typhi organisms and no positive the corresponding DNA were detectable in the blood of the donors. The samples were used in the following procedures. A mid-log-phase culture of *S*. Typhi (OD = 0.6, approximately 10^8^ CFU/mL) was diluted to cover a serial 10-fold gradient from 10^7^ to 10^0^ CFU/mL. One-half of a milliliter of various dilutions of the bacteria was mixed with 3 mL of the fresh anticoagulant blood and incubated at room temperature for 30 min to produce the whole simulated blood specimens[23]. The total DNA was extracted from the simulated blood specimens by the QIAamp DNA Blood Mini Kit (QIAGEN) according to the manufacturer’s protocol. The DNA was eluted in a final volume of 50 μl of DNase-free sterile pure water, and 5 μl of the elution DNA were subjected to both the LAMP and RT-PCR assays, which were repeated twice for each sample. Meanwhile, colony counts using the standard plating method were accomplished to determine an accurate bacterial content in the simulated samples. The human blood spiked with no *S*. Typhi was used as the negative control. This experiment was independently repeated three times.

### An enrichment experiment of simulated blood samples with lower S. Typhi counts

Approximately 2 mL of a blood specimen spiked with 10^0^, 10^1^, or 10^2^ CFU/mL *S*. Typhi (as described above) was injected into each 40 mL blood culture bottle (BD BACTEC lytic/ 10 Anaerobic/F culture Vials) and incubated at 37°C for 10 h. Each group had three parallel experiments. Meanwhile, colony counts using the standard plating method were performed to determine an accurate bacterial count in the anaerobic vials at the beginning. Two milliliters of the culture sample were taken out from the bottle every hour during the 10 h incubation period, and DNA was extracted using the QIAamp DNA Blood Mini Kit (QIAGEN). The STY2879-LAMP and RT-PCR assays were performed to detect the *S*. Typhi pathogen in the blood.

### The preparation of the pathogen-simulated stool specimens and the heat-treatment DNA template

We obtained a healthy donor’s stool specimens and examined the stool specimens by culture assay and PCR [22] to confirm that there was no *S*. Typhi contamination. A mid-log-phase culture of *S*. Typhi (OD = 0.6, approximately 10^8^ CFU/mL) in LB was diluted serially from 10^7^ ~ 10^0^ CFU/mL in physiological saline and quantified using the standard plating method. At the same time, 0.2 mL of the dilution culture was mixed well with 0.2 g feces, and final concentrations of 10^7^ ~ 10^1^ CFU/g of the *S*. Typhi spiked stool samples were prepared. The feces spiked with no *S*. Typhi was the negative control. Five milliliters of Selenite Cystine (SC) broth was added to each aliquot of the spiked stool sample, mixed well and incubated overnight at 37°C. Five hundred microliters of the sample was taken out from every enrichment culture broth at every hour during the incubation processing. Both the pre-enrichment and post-enrichment samples were handled in the following steps to prepare simple heat-treatment of the DNA samples. The samples were centrifuged at 1,000 rpm for 1 min, the supernatant was retained, and the large particle precipitate was discarded. The supernatant was then centrifuged at 8,000 rpm for 5 min, the supernatant was removed, and the precipitant was retained. One milliliter of TE buffer was used to wash the pellets (this step was repeated twice). TE was added to suspend the bacteria pellet, and the sample was boiled for 5 min. The samples were centrifuged at a speed of 12,000 rpm for 5 min, and the crude extract supernatants were used as the template to determine the sensitivity of both the STY2879-LAMP and RT-PCR assays. The experiment was independently repeated three times.

### LAMP assay

**Fig 1:**
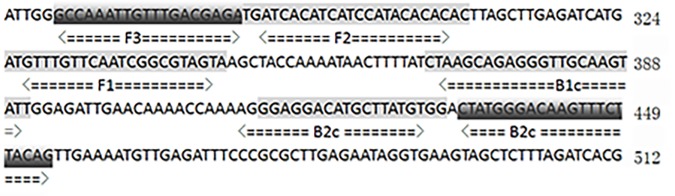
Target sequences of primers for STY2879-LAMP. Nucleotide sequence of STY2879 gene used for designing the inner and outer primers. The outer primers consist of B3 and the sequence (F3) complementary to F3c, respectively. Two inner primers were labeled as forward inner primer (FIP) and the backward inner primer (BIP), respectively, and each contains two distinct sequences corresponding to the sequence and complementary sequence of the target gene.

A set of four primers comprising two outer and two inner primers that recognize six distinct regions in the target STY2879 gene was designed by the LAMP primer designing software PrimerExplorer V4 (Eiken Chemicals, Japan, http://primerexplorer.jp/e/). The forward inner primer (FIP) was composed of the complementary sequence of F1 (F1c) and F2 sequence, and the backward inner primer (BIP) consisted of B1c and B2. Two outer primers F3 and B3 were used for the initiation of the STY2879-LAMP reaction. The sequence of each primer is shown in Table 2 and Fig 1. All LAMP reactions were performed with the Loopamp DNA Amplification Kit (Eiken, Tokyo, Japan) in a 25-μL mixture containing 1.6 mM FIP and BIP primers (each), 0.2 mM F3 and B3 primers (each), 20 mM Tris-HCl (pH 8.8), 10 mM KCl, 8 mM MgSO_4_, 10 mM (NH_4_)_2_SO_4_, 0.1% Tween 20, 0.8 M betaine, 1.4 mM deoxynucleoside triphosphates (dNTPs; each), 1 μL of Bst DNA polymerase (8 U/μL), and 5 μL of the template DNA. One microliter of the Fluorescent Detection Reagent (FDR) was added to each LAMP assay before the reaction, which allows the amplification reaction to be visually detected using an ultraviolet (UV) lamp or the naked eye. The reaction mixtures were set up in tubes and incubated in a Realtime Turbidimeter LA320C (Teramecs, Tokyo, Japan) at 65°C for 60 min, followed by 80°C for 5 min to terminate the reaction. The positive samples were distinguished from the negative samples by a turbidity cutoff value of ≥ 0.1 or by a color switch from the original orange to green. After amplification, the LAMP products were detected by electrophoresis on 2% agarose gels with ethidium bromide staining.

**Table 2:** The primers for STY2879-LAMP and RT-PCR in this study.

Assay	Primer	Genome position	Sequence(5’-3’)	Length
LAMP	F3	266–284	GCCAAATTGTTTGACGAGA	19
	B3	434–455	CTGTAAGAAACTTGTCCCATAG	22
	FIP(F1c-F2)	F1c:325–347F2:285–307	TACTACGCCGATTGAACAAACAT-TGATCACATCATCCATAAACACA	46
	BIP(B1c-B2)	B1c:369–391B2:414–432	CTAAGCAGAGGGTTGCAAGTATT-CCACATAAGCATGTCCTCC	42
RT-PCR	F3	266–284	GCCAAATTGTTTGACGAGA	19
	B3	434–455	CTGTAAGAAACTTGTCCCATAG	22

The genome position according to the *S*. Typhi strain CT-18 (GenBank accession number: GI:30407157).

### The construction of a reference plasmid carrying the STY2879 gene

The reference plasmid pEASY-T1-STY2879 was constructed as follows: the full length STY2879 gene, which was from the *S*. Typhi strain CT18, was amplified by a set of primers, STY2879-F: 5’-3’ ttacaacttaaagagtttcgc and STY2879-R: 5’-3’ atgacaatagaagttcaaagg, and the PCR products (774 bp) were ligated into the pEASY-T1 vector according to the pEASY-T1 Cloning Kit (Transgen, Beijing, China). The recombinant plasmid designated pEASY-T1-STY2879 was confirmed by sequencing. To test the sensitivities of the STY2879-LAMP assay and RT-PCR, we quantified the concentration of the recombinant plasmid by a NanoDrop ND-1000 apparatus (Thermo Scientific) and 10-fold serially diluted the plasmid from 10^5^ to 10^0^ copies/μL to define the limit of detection. The concentration (ng/μL) of the plasmid was converted to the copy number using the formula: mol/g × molecules/mol = molecules/g.

### RT-PCR

To compare the sensitivity and specificity of the STY2879-LAMP assay, conventional RT-PCR was also performed with the primers F3 and B3, shown in Table 2, using the SYBR Premix Ex Taq^TM^ II reagents (TaKaRa, Japan) on the CFX96 real-time platform (C1000TM Thermal cycler, BioRad) with settings of pre-denaturation at 95°C for 30 sec, 39 cycles of denaturation at 95°C for 5 sec, and an extension at 58°C for 30 sec. The fluorescence readings were acquired using the 6-carboxyfluorescein (FAM/SYBR) channel. The RT-PCR results were defined as positive for a Ct less than 37.

## Results

### Target gene screening for *S*. Typhi detection

To obtain the specific genes of *S*. Typhi, we first compared the genes from two available complete genomes of *S*. Typhi CT18 and Ty2 (accession numbers in GenBank: NC_003198 and NC_004631). The genes shared by these two genomes were searched in the genomic sequences of other *Salmonella* serotypes and then compared with the human genome. The genes that could be aligned with genomes of other *Salmonella* strains or the human genome were filtered out, and the remaining ones were considered as the specific genes of *S*. Typhi. Sequence comparisons were performed using the BLAST-Like Alignment Tool with the default parameters. The alignments less than 50% of the gene size were ignored, and a total of 125 genes were obtained. Furthermore, the candidate genes were used as a bait gene to do a BLASTN search in the PubMed database to find the genes only existing in *S*. Typhi. At the same time, to test the specificity of each gene for detecting *S*. Typhi, ordinary PCR was performed with 125 sets of primers (data not shown) and the DNA from 22 *S*. Typhi strains and 75 non-typhoidal *Salmonella* strains as templates (Table 1) for the second round of screening. Finally, we found that the STY2879 gene was a unique gene in *S*. Typhi; no other similar sequence was found in the other organisms, indicating that STY2879 may only exist in *S*. Typhi in the current online database. Therefore, STY2879 was chosen as the target gene for detecting *S*. Typhi. The results showed that 100% positive amplifications of STY2879 were observed in 22 *S*. Typhi strains isolated in different years and regions. No false positive reaction was found in 75 non-typhoidal Typhi strains (data not shown). All of the above assays were performed in triplicate. The primers applied in the STY2879 PCR are the same as those used in the construction of the STY2879 gene reference plasmid. The STY2879 PCR amplicons (774 bp) from 10 *S*. Typhi strains were randomly selected and sequenced to confirm the stability and conservation of the STY2879 gene. By a BLAST comparison, the sequence identity was 100%. These results indicated that STY2879, which has a high conservation and specificity, may be used as a target gene for the development of *S*. Typhi detection.

### The establishment of the STY2879-LAMP assay

Multiple sets of primers based on the STY2879 gene sequence for LAMP were designed by using the Primer Explorer V4 software. We selected six sets of LAMP primers (data not shown) from the all of the software-generated primer sets, according to the primer advantage factors. Although no suitable loop forward (LF) or backward primer (LB) was found, one set of primers (Table 2) could be used in the LAMP reaction within the shortest time (less than 60 min) and performed well in a wide temperature range from 60°C to 65°C. To ensure the specificity of the detection, we set all of the following LAMP temperatures at 65°C for 60 min using the Realtime Turbidimeter LA320C (Teramecs, Tokyo, Japan). The LAMP assay was executed in a final reaction volume of 25 μL, which contained the reaction buffer, Bst DNA polymerase, and four primers, as well as the template from a total DNA extraction of the *S*. Typhi standard strain CT18, as described in Materials and methods.

### The specificity and sensitivity of STY2879-LAMP

Two microliters of the DNA extraction sample (50 ng/uL) from each of 97 bacterial strains (Table 1) were used to evaluate the specificity of the STY2879 gene based on LAMP and RT-PCR. Positive amplifications were obtained in all the *S*. Typhi strains by LAMP within 60 min of the incubation period, whereas negative results were obtained for the other 75 bacteria strains, even within a prolonged 120 min. The same results were obtained with RT-PCR. The results showed that the primer sets (Table 2) used for STY2879-LAMP and RT-PCR are specific to *S*. Typhi.

**Fig 2:**
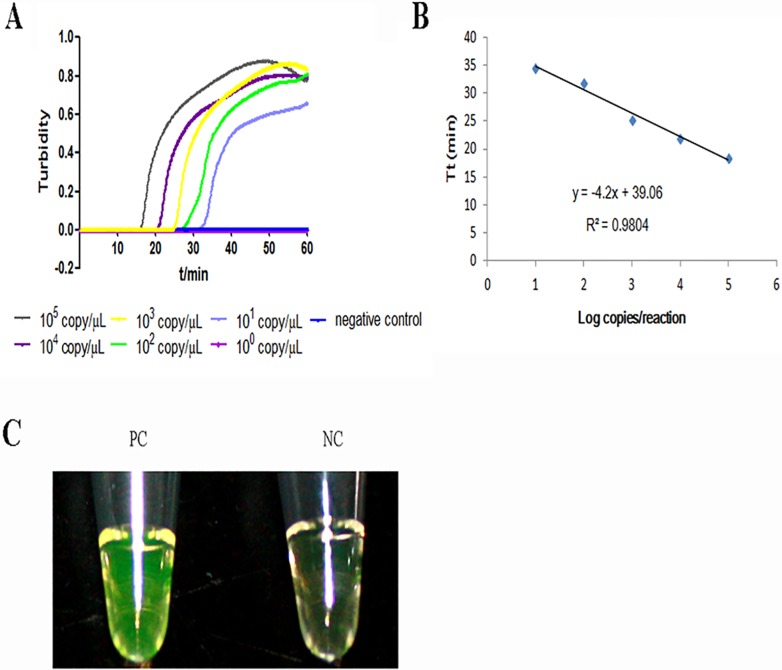
The real-time amplification of the STY2879 gene by LAMP. A. The real-time sensitivity of STY2879-LAMP as monitored by measurement of turbidity (optimal density at 650 nm). A turbidity of ≥ 0.1 was considered to be positive for STY2879-LAMP. Ten-fold serial dilutions of the reference plasmid pEASY-T1-STY2879 ranging from 1.5 × 10^5^ to 10^0^ copies/reaction were tested. B. The relationship between the threshold time (Tt) of each sample and the log copies/reaction. The standard curve was drawn on the basis of three independent repeats and the linear relationship R^2^ = 0.9804. C. A direct visual detection by the naked eye with the addition of the FDR dye, which was added prior to the amplification. The original orange color of the FDR changed to green in the case of a positive amplification under natural light, whereas the original orange color was retained for a negative reaction.

The sensitivity of STY2879-LAMP and RT-PCR was evaluated with the reference plasmid. It showed that the sensitivity of STY2879-LAMP was 15 copies/reaction (Fig 2), which was 10-fold higher when compared to the detection limit of 150 copies/reaction for RT-PCR (data not shown). The linear relationship between the threshold time (Tt) of each sample and the log copies/reaction was R^2^ = 0.9804, indicating the reference plasmid is well-suited for quantitative LAMP detection of STY2879 gene. Because the FDR fluorescent dye was also added to the LAMP system, the results could be directly observed visually after the turbidity was detected by LA320C. We found that the result with the fluorescent dye was consistent with that of the turbidity test (data not shown). This finding indicates that the two detection methods for sensitivity analysis are feasible.

### An evaluation of the STY2879-LAMP assay with the simulated human stool samples

**Fig 3:**
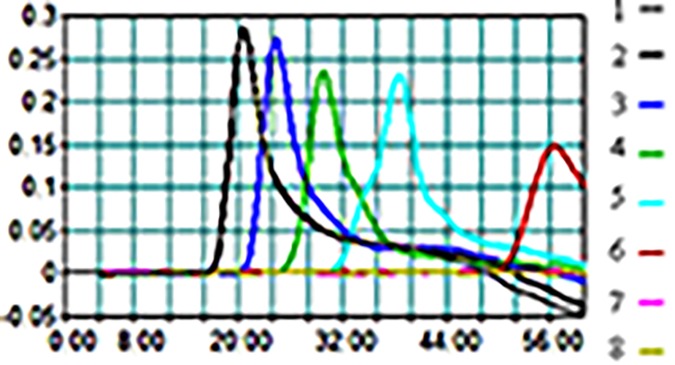
The amplification of STY2879-LAMP in detecting *S*. Typhi in simulated human stool samples. The assay was performed using simple heat-treated DNA samples from pre-enrichment simulation stool specimens spiked with different levels of *S*. Typhi (CT18) ranging from 2×10^7^～2×10^1^ CFU/g (sample 1–7); 8 is the negative control without the target DNA. The amplification curves of sample 1 and 2 are overlapping.

The heat-treated DNA samples from the simulated human stool samples (pre-enrichment) were analyzed by STY2879-LAMP. At the same time, the same amount of the DNA samples was tested by RT-PCR as well. As the results in Fig 3 show, when the reaction time was close to 60 min, the sensitivity of STY2879-LAMP reached 2×10^2^ CFU/g, and the detection limit of RT-PCR was 2×10^4^ CFU/g in terms of the same simulated stool samples (data not shown). Meanwhile, we enriched the simulated human stool samples spiked with a low level of 2×10^1^～10^0^ CFU/g in SC broth for further analysis. The results of STY2879-LAMP and RT-PCR are summarized in Table 3 for the samples spiked with two low levels of *S*. Typhi strains after various enrichment periods. None of the samples (2×10^1^～10^0^ CFU/g) enriched for 3 h was tested positive for *S*. Typhi by either STY2879-LAMP or RT-PCR. We observed positive results with STY2879-LAMP at the 4 h to 5 h enrichment time point, while the positive detection by RT-PCR was delayed for 3 hours. The positive detection time of the simulated human stool samples with 2×10^1^ CFU/g *S*. Typhi was one hour earlier than that of the samples spiked with 2×10^0^ CFU/g *S*. Typhi by either STY2879-LAMP or RT-PCR. These data showed that for those samples with a lower concentration of *S*. Typhi (< 2 ×10^2^ CFU/g), it will take approximately 4–5 h to enrich the original sample. The STY2879-LAMP assay combined with customary culture methods may shorten the detection time compared to the culture method.

**Table 3:** A comparison of the STY2879-LAMP and RT-PCR assays in the human stool samples spiked with two low levels of *S*. Typhi.

Assay	Bacterial concentration (CFU/g)	Enrichment time (hours)										
		0	1	2	3	4	5	6	7	8	9	10
LAMP Tt (min)	2	－	－	－	－	－	35.2	32.5	30.3	27.4	25.1	20.8
	20	－	－	－	－	32.2	30.1	28.0	25.7	22.1	19.4	16.2
RT-PCR Ct (cycles)	2	－	－	－	－	－	－	－	－	35.5	31.0	27.2
	20	－	－	－	－	－	－	－	36.8	31.3	27.3	26.4

“－” means that a positive result with this sample is not available.

### An evaluation of the STY2879-LAMP assay in the simulated human blood samples

**Fig 4:**
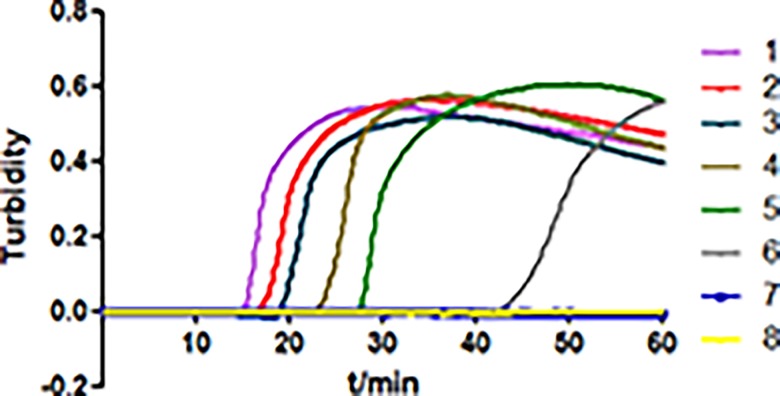
The sensitivity of the STY2879-LAMP assay in human simulated blood samples. The STY2879-LAMP was performed using simulated blood samples spiked with serially diluted *S*. Typhi strain CT18. The sensitivity of the STY2879-LAMP assay was monitored by a real-time measurement of turbidity. The corresponding curves of decreasing concentrations of CT18 are shown from sample 1 to 7 (1–7: from 2×10^6^–2× 10^0^ CFU/mL); 8 is the negative control.

To determine the detection limit of STY2879-LAMP in the human blood specimens, the human blood samples were spiked with different amounts of S. Typhi (2×10^7^–2×10^0^ CFU/mL) to simulate the clinical blood samples. DNA was extracted from each 3 mL simulated blood sample and used as the template of STY2879-LAMP and RT-PCR. A positive amplification by STY2879-LAMP was observed in the 20 CFU/mL sample within one hour (Fig 4), compared to RT-PCR, with a detection limit of 200 CFU/mL in the sample (data not shown). No amplification was obtained in the negative control, which contained only human blood. The evidently positive amplification could be seen within 30 min for those samples with an S. Typhi concentration ≥ 200 CFU/mL. A previous study showed that the median S. Typhi count in the blood of a patient was 1 CFU/mL (with a range of < 0.3 to 387 CFU/mL) [24]; therefore, we enriched the blood samples spiked with lower S. Typhi counts (10^–1^, 10^0^, 10^1^ CFU/mL levels of the samples) with blood culture bottles incubated at 37°C for 10 h. Two milliliters of the enrichment sample were removed every hour for DNA extraction. As shown in Table 4, 1–3 hours enrichment of the simulated blood samples with 10^1^, 10^0^, or 10^–1^ CFU/mL of S. Typhi were needed to obtain a positive detection by STY2879-LAMP; a longer incubation time (3–6 h) of the samples was needed to obtain a positive detection for RT-PCR. Thus, by comparing with RT-PCR, the positive reporting time of STY2879-LAMP was under 3–5 hours. The bacteria number could reach 50 CFU / mL after 3 hours enrichment time by determination the growth curves of the simulated blood samples with 10^–1^ CFU / mL of S. Typhi (S1 Fig), which is enough to reach the detection limit of STY2879-LAMP (Fig 4) and consistent with 3 hours monitoring results (Table 4).

**Table 4:** A comparison of the LAMP and RT-PCR assays in human blood samples spiked with low levels of *S*. Typhi.

Assay	Bacterial concentration (CFU/mL)	Enrichment time (hour)										
		0	1	2	3	4	5	6	7	8	9	10
LAMP Tt (min)	0.1	－	－	－	54.2	52.8	45.6	33.5	30.3	27.4	25.1	20.8
	1	－	－	48.8	42.5	33.2	28.5	25.5	23.2	19.1	18.4	18.2
	10	－	52.5	34.1	30.4	27.4	25.0	21.1	17.6	15.4	15.2	14.8
RT-PCR CT (cycles)	0.1	－	－	－	－	－	－	－	－	35.0	31.7	25.0
	1	－	－	－	－	－	－	34.6	32.8	29.4	27.0	26.1
	10	－	－	－	－	33.5	26.9	23.7	21.5	18.8	16.5	15.3

“－” indicates that a positive result with this sample is not available.

## Discussion

During the past 20 years, high incidence of typhoid and paratyphoid fever has been noted in some regions and outbreaks in these regions are not uncommon. Due to the similarities in systemic symptoms caused by typhoid and paratyphoid, it is hard to distinguish them from each other; in additon it is also difficult to differentiate typhoid from other febrile illness in early phase of the disease. Many patients with typhoid do not have the opportunity to obtain a rapid and reliable diagnosis because of a lack of diagnosis settings and technical personnel in economically poor areas [5, 25]. Therefore, the general lack of etiological diagnosis of typhoid results in the persistence and accumulation of patients and carriers which to some extent attributes to the outbreak and high incidence of typhoid fever, demanding the necessity of developing a rapid, accurate and cost-effective method applied in developing counties especially in rural areas with high incidence of typhoid.

Though some rapid nucleic acid amplification technologies such as multiplex PCR, RT-PCR and LAMP have been developed to detect *S*. Typhi based on some virulence genes [14–18], cross reactions were observed, for example, *fliC*-LAMP has cross reactions with the *S*. Schwarzengrund, *S*. Livingstone, *S*. Liverpool, and *S*. Stanly strains [26]. Although the *invA* gene was used in LAMP to detect *S*. Typhi [27], similar sequences of the *invA* gene can be found in *S*. Typhimurium (*invA*, with 99.7% identity) and *S*. *flexneri* (*virH*, with 64.3% identity). Some genes in pathogenic island used as target of PCR for *S*. Typhi detection[28–31] such as viaB were found to have lost in some *S*. Typhi isolates in recent years [32, 33]. Therefore, identifying the specific and conservative genes in pathogenic microorganisms is crucial for diagnosis using nucleic acid amplification technology. In our study, we adopted a two-rounds screening strategy to ensure the specificity of the target gene of *S*. Typhi. After gene blasting and PCR confirmation we found that the STY2879 gene of *S*. Typhi was very conservative, with 100% sequence identity in 20 *S*. Typhi strains isolated from different years and regions, but no similar genes were found in other vast pathogens, making this gene suitable as a molecular target employed in LAMP detection of *S*. Typhi infection.

*S*. Typhi gene STY2879 encodes a reverse transcriptase family protein with 257 aa which shows 99% similarity to RNA-directed DNA polymerase (WP_000151541) from *Escherichia coli* by NCBI blast, but the nucleotide sequence is specific in *S*. Typhi. The main function of STY2879 and its contribution to the phenotype or pathogenesis of *S*.Typhi may be explored in the future. But it does not affect its use as a target gene to detect S. Typhi as demonstrated by our results. In our study, we used a reference plasmid containing STY2879 to analyze the amplification efficiency of designed primers. The use of reference plasmid provides us a relatively simple conditions under which we can exactly, stably and quantitatively analyze the sensitivity and amplification efficiency of the primer of LAMP.

Generally, nucleotide-based detection methods such as PCR and LAMP are subjected to various inhibitors present in clinical samples particularly in stool samples. We found that the sensitivity of the direct heat-treatment DNA template from simulation stool sample was ten times lower than that from pure bacteria (data not shown). Some researchers have reported that the Bst polymerase in LAMP is more tolerable to the inhibitors than Taq polymerase in the amplification of the raw samples [34]. In order to avoid the influence of matrix, we tried to remove the matrix by centrifugation and washing the stool samples and prepared the direct heat-treatment DNA template. Meanwhile, we prepared the direct heat-treatment DNA template from the same volume *S*. Typhi dilution culture (before the simulation stool sample preparation) in the same manner as we dealt with the simulated stool specimens. Our results showed that the LAMP assay was more accurate and sensitive than RT-PCR methods using simulated human blood and stool samples and proved markedly faster than RT-PCR.

Considering a low blood load of *S*. Typhi, it is better to perform enrichment before the LAMP assay. It is time consuming if we perfomed LAMP assay combining culture, however, the whole detection procedure would be completed within one day, as compared to at least 3 days for culture alone, thereby significantly shortening the total assay time. For stool sample, the detection limit of STY2879-LAMP was low enough to detect *S*. Typhi from stool (the 500 CFU/g limitation for culture); when performed in combination with enrichment, it would dramatically dilute the inhibitors and increase the effectiveness of LAMP assay, especially when low amount of pathogens survived post antibiotics administration.

It should be noted that the cost of LAMP assay is higher than the available rapid diagnostic techniques such as lateral flow, Widal test, and PCR, but the sensitivity of LAMP is much higher than lateral flow [7], Widal test needs skilled technician and PCR procedure is complicated. Another disadvantage of STY2879-LAMP assay is that DNA extraction from simulated blood samples was required for STY2879-LAMP assay because the direct boil-ruptured blood cells had a red color that may influence the final correct judgment of the fluorescence. Further studies can be performed to analyze the impact of the red color from blood on the turbidity measurement or to devise a method remove the color. For example, the pathogenic nucleotide or the whole nucleotide could be isolated from the sample using magnetic beads to avoid the color interference. Meanwhile, the method could increase the detection limit of the LAMP assay or RT-PCR amplification.

In conclusion, we established a LAMP assay based on the STY2879 gene of *S*. Typhi with a high specificity and sensitivity. Through the laboratory estimation of three different samples, including a quantitative reference plasmid, simulated stool and blood, STY2879-LAMP exhibits an obviously higher sensitivity than RT-PCR in detection of *S*. Typhi in these samples, highlighting its potential in molecular diagnosis of typhoid fever by identifying the positive samples for further culture and isolation of *S*. Typhi, as well as for rapidly reporting more sensitive results. This study also shows the feasibility of the practical assessment of STY2879-LAMP in the clinical laboratory and field laboratory, though it needs vast number of clinical samples to validate its effectiveness.

## Supporting Information

S1 Fig The growth curves of the simulated blood samples with 10^–1^ CFU / mL of *S*. Typhi. The simulated blood samples was injected into each 40 mL blood culture bottle (BD BACTEC lytic/ 10 Anaerobic/F culture Vials) to make the final culture with 10^–1^ CFU / mL of *S*. Typhi and incubated at 37°C for 14 h. Throughout the enrichment process, the growth curve was plotted by counting the bacteria number and determining the concentration of bacteria at every hour.
(TIF)
