# Supplementary figures and images for: The Development and Evaluation of a Loop-Mediated Isothermal Amplification Method for the Rapid Detection of Salmonella enterica serovar Typhi
Source: PLoS One. 2015 Apr 24;10(4):e0124507. doi: 10.1371/journal.pone.0124507 (PMC4409374; doi:10.1371/journal.pone.0124507)

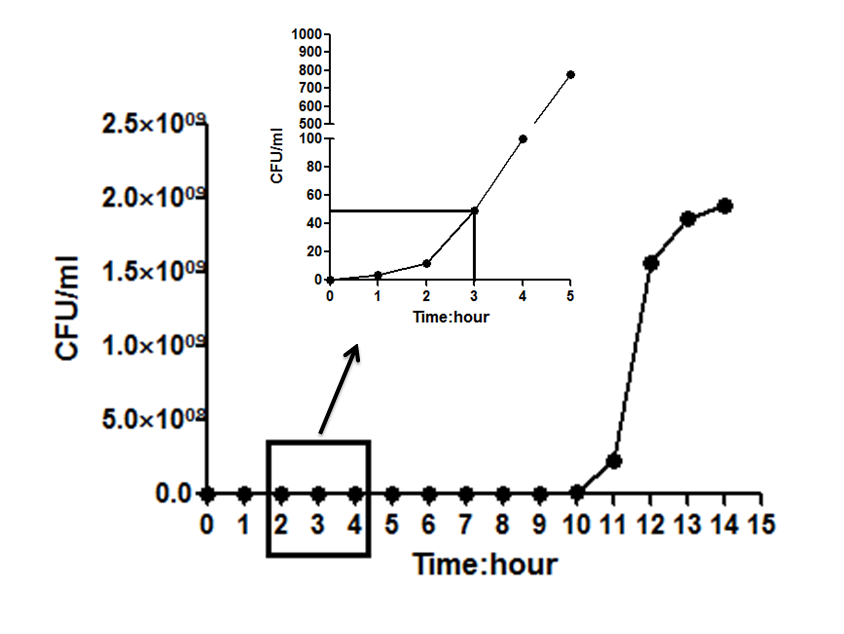

Supplement: S1 Fig — The simulated blood samples was injected into each 40 mL blood culture bottle (BD BACTEC lytic/ 10 Anaerobic/F culture Vials) to make the final culture with 10–1 CFU / mL of S. Typhi and incubated at 37°C for 14 h. Throughout the enrichment process, the growth curve was plotted by counting the bacteria number and determining the concentration of bacteria at every hour. (TIF) [file pone.0124507.s001.tif]
